# The data on the relationship between polymorphism of HTR1B and DBH genes and attention-deficit hyperactivity disorder in adults with or without substance use disorders

**DOI:** 10.1016/j.dib.2018.06.098

**Published:** 2018-07-03

**Authors:** Mojgan Khademi, Katayoon Razjouian, Rozita Davari-Ashtiani, Fariba Arabgol, Firoozeh Jafari, Hosein Darvish

**Affiliations:** a Child and Adolescent Psychiatry, Shahid Beheshti University of Medical Sciences, Tehran, Iran; b Behavioral Sciences Research Center, Shahid Beheshti University of Medical Sciences, Tehran, Iran; c Child and Adolescent Psychiatrist in Shahid Beheshti University of Medical Sciences, Tehran, Iran; d Department of Medical Genetics, School of Medicine, Shahid Beheshti University of Medical Sciences, Tehran, Iran

**Keywords:** Attention-deficit hyperactivity disorder, Polymorphism, HTR1B gene, DBH gene, Substance use disorders

## Abstract

There is a positive relationship between attention-deficit hyperactivity disorder and tendency toward drug use in numerous studies. The present study was aimed to investigate the relationship between polymorphism of serotonin receptor 1B gene (HTR1B) and Dopamine beta-hydroxylase gene (DBH) with attention-deficit hyperactivity disorder in adults with or without substance use disorders. In the present case-control study, as many as 355 individuals entered the present study and was categorized in different groups: control healthy group, substance use disorders group, and attention-deficit hyperactivity disorder group. For confirming attention-deficit hyperactivity disorder in adults, demographic and Conners forms were used. Moreover, SCID-I questionnaire was used to confirm or reject the individual׳s suffering from substance use disorders and other psychiatric diseases. The polymorphism of abovementioned genes was conducted by polymerase chain reaction (PCR) and restriction fragment length polymorphism (RFLP). In case of DBH gene-Rs2519152, the findings indicated that TT, TC, and CC genotypes and T and C alleles are not different in the attention-deficit hyperactivity disorder group, substance use disorder group, the group with patients suffering from both disorders, and control group. Moreover, the frequency of TT, TA, and AA genotypes as well as T and A alleles was same in the attention-deficit hyperactivity disorder group, substance use disorder group, the group with patients suffering from both disorders, and control group.

## Specifications Table

**Table:** 

Subject area	Genetic
More specific subject area	Medical genetic
Type of data	Tables
How data was acquired	In this case-control study, the questionnaire and experimental data was collected from 355 individuals, which was grouped into 3 groups, including control healthy group, substance use disorders group, and attention-deficit hyperactivity disorder group.
Data format	Raw and analyzed
Experimental factors	The polymorphism of abovementioned genes was done by polymerase chain reaction (PCR) and restriction fragment length polymorphism (RFLP).
Experimental features	All the experiments carried out in this study was in accordance to standard methods previously reported in the valid studies.
Data source location	Tehran, Tehran province, Iran.
Data accessibility	Data are included in this article

## Value of the data

**Table:** 

• Attention-deficit hyperactivity disorder is known as one of the nervous disorders prevalent in children and teenagers and continues to adulthood [1]. The global prevalence of attention-deficit hyperactivity disorder is 4%, and the most important cause of this disease is the genetic factors [1], [2], [3]. • The relationship between attention-deficit hyperactivity disorder and HTR1B and DBH genes has not studied before. • The data in this article indicates that genotype and allele frequency difference of HTR1B (rs130058) and DBH (rs2519152) genes are not different in the attention-deficit hyperactivity disorder group, substance use disorder group, the group with patients suffering from both disorders, and control group. • This data shows that no correlation was observed between polymorphism of DBH and HTR1B genes with attention-deficit hyperactivity disorder and substance use disorders either separately or simultaneously. • The data in this study is interesting in comparison to other studies, due to the use of different genes and SNPs in different races.

## 1 Data

Table 1 presents the distribution and genotype frequency of HTR1B and DBH genes in different groups. In addition, Table 2 shows the distribution and allele frequency of HTR1B and DBH genes in different groups investigated. The findings indicated that genotype and allele frequency difference of HTR1B (rs130058) and DBH (rs2519152) genes are not different in the attention-deficit hyperactivity disorder group, substance use disorder group, the group with patients suffering from both disorders, and control group (Table 1, Table 2). Thus, there is no relationship between the polymorphism of the abovementioned genes with attention-deficit hyperactivity disorder, substance use disorder, and both disorders at the same time.

**Table 1:** The distribution and genotype frequency of HTR1B and DBH genes in different groups investigated.

**SNP**	**Gene**	**Groups**	**Genotypes frequencies**, *n***(%)**		
			**TT**	**TC**	**CC**
Rs2519152	DBH	Control	29(30)	50(52)	17(18)
		SUD	24(23)	64(60)	18(17)
		ADHD	13(34)	18(47)	7(19)
		ADHD + SUD	15 (24)	38(61)	9(15)
			TT	AT	AA
rs130058	HTR1B	Control	41(43)	43(45)	12(12)
		SUD	32(30)	59(56)	15(14)
		ADHD	14(37)	21(55)	3(8)
		ADH + SUD	20 (32)	34(55)	8(13)

**Table 2:** The distribution and allele frequency of HTR1B and DBH genes in different groups investigated.

**SNP**	**Gene**	**Groups**	**Allele frequencies, *n* (%)**		**Odds ratio (95%CI)**		
			**T**	**C**	**Control**	**Addiction**	**ADHD**
Rs2519152	DBH	Control	108(56)	84(44)	–	0.87 (0.58–1.2)	1.06 (0.62–1.83)
		SUD	112(47)	100(53)	0.87 (0.58–1.2)	–	1.22 (0.72–2.08)
		ADHD	44(58)	32(42)	1.06 (0.62–1.83)	1.22 (0.72–2.08)	1
		ADHD + SUD	68(55)	56(45)	0.94 (0.59–1.48)	1.08 (0.69–1.69)	0.88 (0.49–1.57)
			*T*	*A*	Control	Addiction	ADHD
rs130058	HTR1B	Control	125(65)	67(35)	–	0.74 (0.49–1.10)	0.97 (0.55–1.69)
		SUD	123(58)	89 (42)	0.74 (0.49–1.10)	–	1.31 (0.76–2.26)
		ADHD	49(64)	27(36)	0.97 (0.55–1.69)	1.31 (0.76–2.26)	–
		ADHD + SUD	74(60)	50(40)	0.79 (0.49–1.26)	1.07 (0.68–1.68)	0.81 (0.45–1.47)

## 2 Experimental design, materials and methods

In the present case-control study, as many as 355 individuals were selected from the patients referred to Imam Hussein Hospital and patients hospitalized at residential centers of Rebirth Charity Society; control healthy group (individuals who had referred to the laboratory of Imam Hussein Hospital, substance use disorders group, and attention-deficit hyperactivity disorder group). Demographic as well as Conners forms were filled out by the participants. Then a clinical-psychiatric interview was conducted to confirm adulthood attention-deficit hyperactivity disorder. The confirmation or rejection of suffering from substance use and other psychiatric diseases was conducted based on SCID-I questionnaire. For the initial diagnosis of attention-deficit hyperactivity disorder, the Conners score of 55 and higher was determined. The present study was conducted under the supervision of Ethics Committee on Research of Shahid Beheshti University of Medical Sciences. After obtaining a written and oral consent from the individuals having the inclusion criteria, as much as 5 cc of blood was taken by using EDTA tubes, and these samples were sent to the Genetics Department of the Faculty for conducting the tests needed.

Genomic DNA extraction of blood samples was conducted by salting out method. The investigation of polymorphism of specific regions was conducted by using specific primers and based on PCR-RFLP method. The sequence of specific forward and reverse primers were 5-AGGACAGGCTCATCCCTCTT-3 and 5-GAGATAATCCTGGAAGCAATCG-3 respectively. For conducting polymerase chain reaction (PCR), the reaction mixture with the final volume of 52 μl was prepared in Eppendorf tubes with the volume of 200 ml according to the Table 3.

**Table 3:** The components used in each PCR for M235T polymorphism.

**Reactive materials**	**Volume (ml)**
Forward Primer	1.25
Reverse Primer	1.25
dNTPs (10 Mm)	0.5
MgCl2 (50 Mm)	0.75
buffer (10 X)	2.5
Taq DNA polymerase (5 u/μl)	0.2
ddH2O	17.5
Genomic DNA	1

After adding sterile mineral oil to the mixture, the tubes were centrifuged, and then they were transferred to the thermal cycler. Initial Denaturation: 94 °C for 5 min, 30 cycles of: denaturation: 94 °C for 30 s, annealing: 53 °C for 30 s, extension: 72 °C for 30 s, final extension: 72 °C for 7 min. In the last stage, the digested products were electrophoresed in 3% agarose gel. After being dyed with ethidium bromide, the gel was placed in UV apparatus, and UV radiation was conducted to observe the bands.

Data handling and descriptive statistics were done using SPSS 21.
